# Inorganic Nanoparticles in Bone Healing Applications

**DOI:** 10.3390/pharmaceutics14040770

**Published:** 2022-03-31

**Authors:** Alexandra-Cristina Burdușel, Oana Gherasim, Ecaterina Andronescu, Alexandru Mihai Grumezescu, Anton Ficai

**Affiliations:** 1 Department of Science and Engineering of Oxide Materials and Nanomaterials, Faculty of Applied Chemistry and Materials Science, University Politehnica of Bucharest, 1–7 Gheorghe Polizu Street, 011061 Bucharest, Romania; alexandra_burdu@yahoo.com.sg (A.-C.B.); oana.gherasim@inflpr.ro (O.G.); agrumezescu@upb.ro (A.M.G.); anton.ficai@upb.ro (A.F.); 2 Lasers Department, National Institute for Lasers, Plasma and Radiation Physics, 409 Atomiștilor Street, 077125 Magurele, Romania; 3 Academy of Romanian Scientists, 3 Ilfov Street, 050044 Bucharest, Romania; 4 Research Institute of the University of Bucharest—ICUB, University of Bucharest, 90–92 Panduri Road, 050657 Bucharest, Romania

**Keywords:** bone regeneration, inorganic nanoparticles, bioceramic nanoparticles, oxide nanoparticles, metallic nanoparticles

## Abstract

Modern biomedicine aims to develop integrated solutions that use medical, biotechnological, materials science, and engineering concepts to create functional alternatives for the specific, selective, and accurate management of medical conditions. In the particular case of tissue engineering, designing a model that simulates all tissue qualities and fulfills all tissue requirements is a continuous challenge in the field of bone regeneration. The therapeutic protocols used for bone healing applications are limited by the hierarchical nature and extensive vascularization of osseous tissue, especially in large bone lesions. In this regard, nanotechnology paves the way for a new era in bone treatment, repair and regeneration, by enabling the fabrication of complex nanostructures that are similar to those found in the natural bone and which exhibit multifunctional bioactivity. This review aims to lay out the tremendous outcomes of using inorganic nanoparticles in bone healing applications, including bone repair and regeneration, and modern therapeutic strategies for bone-related pathologies.

## 1. Introduction

Bone is a dynamic tissue that is constantly renewed and repaired through its intrinsic remodeling process, which involves interactions between resident cells (osteoclasts and osteoblasts) and signaling factors, that remove old and damaged tissue and create new bone, respectively [1,2]. This fine-tuned synergy is responsible for the preservation of bone balance. The healing of bone fractures and the restoration of critical bone anomalies are difficult tasks for orthopedics, traumatologists, and maxillofacial surgeons [3]. Given the specific patient-related requirements and limitations in bone regeneration, the clinical use of synthetic bone substitutes represents one of the most important updates in bone regenerative therapy [4,5]. The current progress in nanotechnology-derived biomaterials enables the development of bone implants that are osteoconductive and osteoinductive, as well as biocompatible, biodegradable, and bioresorbable [6,7,8].

Nanobiomaterials include nanometer-sized and nanostructured bioactive materials, which peculiar behavior and new properties strongly impact the emerging trends of modern biomedicine and biotechnology [9,10]. Nanostructured biomaterials possess improved and superior bone regeneration ability thanks to their particular physicochemical properties and biological behavior, which are quite different from their bulk counterparts [11,12]. During the last decade, various nanoparticle-based protocols have been successfully evaluated for the diagnosis and targeted treatment of orthotopic and metastatic bone cancers [13,14]. The size of nanoparticles (NPs, 1–100 nm size range) permits their passage through biological barriers, while their size-related features (including a high surface area-to-volume ratio, surface energy and reactivity, mechanical, thermal, optical, electrical and magnetic properties governed by quantum effects, and intrinsic biological activity) enable them to attain significant therapeutic efficacy [15,16]. Moreover, nanoengineered platforms may increase drug solubility and improve drug bioavailability, but also enhance pharmacokinetics and pharmacodynamics, and provide specific and selective targeted and/or controlled therapeutic effects [17,18].

With the aim to overcome the drawbacks of classical restorative and replacement procedures of hard tissues (including herein the limited bioavailability and increased immunogenicity of autografts and allografts, but also the bioinertness and limited bioactivity of clinically approved biomaterials) [19,20], an impressive amount of progress has been reported in the development of bone regeneration materials during the last few decades. Biomaterials for hard tissue engineering applications include the following categories: (i) first-generation biomaterials—prosthetic devices made from biologically inert materials, such as metals and alloys, certain synthetic polymers, and bioceramics; (ii) second-generation biomaterials—osteoconductive and osteoinductive devices made from bioactive, biodegradable, and bioresorbable materials, such as calcium phosphates, bioactive glasses, and polyesters; and (iii) third-generation biomaterials—advanced and multifunctional biomaterials with osteogenic properties and the ability to regulate the body’s functions [21,22,23].

As the size-related behavior of NPs is also responsible for the occurrence of circumstantial toxic effects, a real challenge consists in maximizing their therapeutic effects by properly tuning the biocompatibility/multifunctionality balance. Nanosized particles can invade surrounding cells or tissues, and they frequently cluster or migrate inside blood vessels, causing additional damage to distant tissues or organs [24,25]. The toxicity of nanoparticles is determined by various parameters, including shape, size, composition, porosity, surface chemistry and coating, but other factors—such as the aggregation state and interactions with biomolecules—may influence their toxicity in humans [26,27].

Nanoparticle-based delivery systems have many advantages over conventional pharmaceutical formulations. These include reduced side effects, enhanced therapeutic effects, prolonged circulation half-life, improved permeability, and patient compliance [28,29]. Designing and developing performance-enhanced platforms for targeted or non-targeted drug delivery generally implies the precise selection of the nanocarrier, which can be (i) inorganic, including quantum dots (semiconductor-based nanoparticles), metallic (noble metals) and oxide nanoparticles, or (ii) organic, including carbon-based nanostructures, such as polymers, dendrimers, exosomes, micelles, liposomes, and solid lipid NPs [30,31].

Thanks to their high surface reactivity, unique surface physics and chemistry, increased chemical stability and photostability, facile surface modification, quantum yields, improved bioavailability, reduced or absent intrinsic toxicity, extended lifetime, great drug-loading capacity, and controlled drug release ability, inorganic NPs have indisputable advantages as active therapeutic carriers [32,33]. Moreover, by coating the inorganic NPs with additional surface ligands (i.e., proteins, peptides, carbohydrates, etc.), higher reactivity and enhanced functionality can be achieved [34,35]. In general, nanocarriers based on inorganic NPs consist of an inorganic core (metal-/oxide-based nanostructures) and an organic shell (carbon-based compounds, which serve as substrates for bio-macromolecular conjugation and/or as shields that protect the inner core from undesirable physicochemical interactions with the biological microenvironment) [36,37]. Biocompatible nanomaterials based on pristine and metal-doped calcium phosphates [38,39,40], bioceramics [41,42] and vitroceramics [43,44], oxides (such as alumina, ceria, silica, titania, and zirconia) [45,46,47,48,49], and metallic nanostructures [50,51,52] are extensively investigated for the unconventional management of bone tissue injuries.

This review aims to point out the significance of inorganic nanoparticles in bone healing by including relevant and recent data on the NP-based repair and regeneration of bone tissue.

## 2. Bioceramic Nanoparticles

### 2.1. Hydroxyapatite

The conventional therapeutic strategy in bone grafting mainly includes the use of allografts and autogenous grafts, and also different isolated or combined substitutes based on calcium phosphate (CaP) materials [53,54]. CaP-based nanoparticles have been extensively investigated in preclinical and clinical studies as bone graft alternatives [55,56]. The use of CaP nanoparticles can be expanded towards cell-/tissue-specific drug delivery platforms owing to their intrinsic features, such as unique biocompatibility and bioactivity, high adsorptive capacity, composition-/microstructure-related tunable properties, and application-related adjustable biodegradability [57,58].

Particularly successful and promising outcomes in designing biomaterials for hard tissue repair and replacement are related to synthetic hydroxyapatite (HA), Ca_10_(PO_4_)_6_(OH)_2_ [58,59]. Naturally, HA is present in metamorphic and igneous rocks as a natural mineral, but it is also present in teeth and bones as the major inorganic component [60,61]. Tremendous interest has been lately oriented towards the revaluation of naturally-derived HA, which can be extracted from sustainable biogenic sources or wastes [62,63,64,65]. Representative sources for extracting natural HA include: (i) mammalian sources—bovine [66,67,68], ovine [69,70], and swine [71,72] bones; (ii) marine or aquatic sources—fish bones [72,73,74], cuttlefish bones [75,76], and corals [77,78]; (iii) shells—cockle shell [79,80], clam shell [81,82], mussel shell [83,84], snail shell [85,86], and egg shell [87,88]; and (iv) mineral sources [89,90].

**Figure 1:**
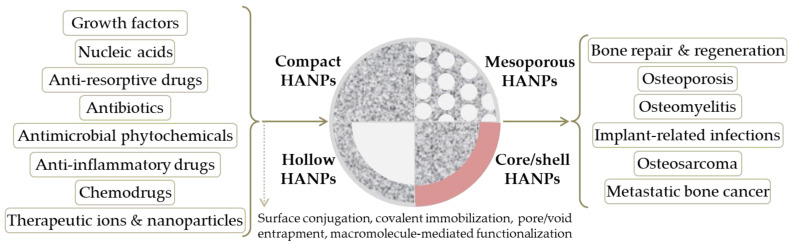
Schematic representation of hydroxyapatite nanoparticles (HANPs) in bone healing applications.

Nanosized HA particles have more unique properties than micro-sized HA particles. For example, it has been reported that nanosized HA exhibits greater protein adsorption, improved cell adhesion, and superior bioactivity when compared to micro-sized HA [60,91]. It also possesses a significant capability to decrease apoptotic death in healthy cells and, therefore, improve cell proliferation and cell activity related to bone growth [91,92]. Given their compositional similarity with the natural bone tissue and their ability to increase new bone formation [93,94,95], HA nanoparticles (HANPs) are regarded as safe candidates for bone-targeted therapy, as summarized in Figure 1.

Possessing excellent biocompatibility and being highly bioactive and biodegradable, HA is widely used for orthopedic, dental, and maxillofacial applications, especially thanks to the unique features of HANPs, which include anti-tumor activity and drug/gene delivery potential [96,97,98]. Even though the intrinsic biocompatibility of nano-hydroxyapatite has been extensively confirmed, recent studies have argued that a thorough screening of HANPs’ toxicity should be conducted to assess their biological effects, as the potential biotoxicity of HANPs (affected by particle diameter, exposure dose, and contact method) was reported [91,99].

**Figure 2:**
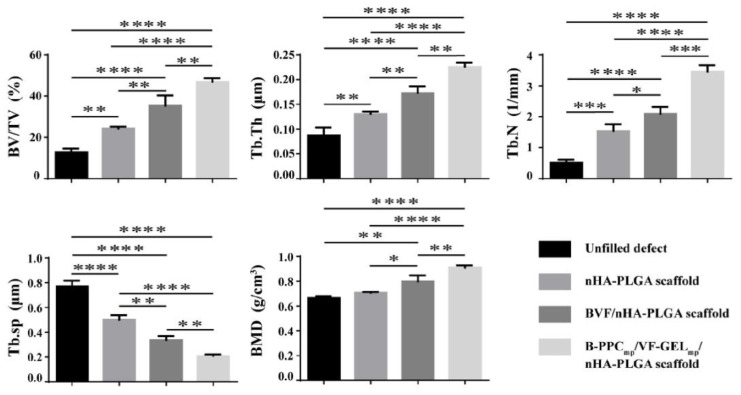
Quantitative representation of bone regeneration induced in rat femur defects by bare nano-hydroxyapatite/poly(lactide-co-glycolide) scaffolds (nHA-PLGA), nHA/PLGA scaffolds modified with BMP-2, VEGF, and FGF-2 (BVF/nHA-PLGA), and nHA/PLGA scaffolds modified with BMP-2-loaded poly(lactic-co-glycolic acid)-poly(ethylene glycol)-carboxyl microparticles and VEGF/FGF-2-loaded gelatin microparticles (B-PPC_mp_/VF-GEL_mp_/nHA-PLGA), evidenced at 12 weeks post-implantation by bone volume fractions (BV/TV), trabecular thickness (Tb.Th), trabecular number (Tb.*n*), trabecular spacing (Tb.Sp), and bone mineral density (BMD). Each data point represents the mean ± standard deviation (*n* = 3), and statistically significant differences are indicated as ∗ *p* < 0.05, ∗∗ *p* < 0.01, ∗∗∗ *p* < 0.001, and ∗∗∗∗ *p* < 0.0001. See Ref. [117]. Reprinted from an open access source.

Although HA is considered to be a suitable material for bone tissue repair and regeneration, its osteoinductive qualities are insufficient to allow large bone defects to mend. To circumvent these drawbacks, several bioactive compounds including growth factors that play a key role during the bone remodeling process, have been employed in bone tissue engineering [100,101,102,103,104]. Osteoinductive growth factors have been utilized in restorative and regenerative procedures for dental [7,105] and orthopedic (craniofacial, spinal fusion and non-union deformities) [54,106,107] pathologies, either alone or combined with ceramic and polymeric or composite materials, with little indication that they are superior to autografts. Bone morphogenetic protein-2 (BMP-2) is the *gold standard* growth factor for enhancing bone healing, and it has been successfully used in various research studies. In terms of osteogenic activity and augmented bone healing, superior results were reported for BMP-2-modified nanostructured formulations based on HA/natural polymers [108,109] and HA/synthetic polymers [110,111]. However, due to its short half-life in vivo, the clinical applicability of BMP-2 is limited, as a suitable BMP-2-loaded bone substitute should accurately provide initial large doses and subsequent constant therapeutic concentrations [112]. Promising HANP-based formulations for orthopedic and orthodontic applications have also been developed via modification with other bone morphogenetic proteins (BMPs) [113,114], fibroblast growth factor (FGF) [101,115], and vascular endothelial growth factor (VEGF) [116,117] (Figure 2), which beneficially contribute to bone matrix mineralization, osteoblastogenesis and new bone formation, implant osteointegration, and vascularization.

The synergistic efficacy of HANPs coupled with anti-osteoporotic compounds has been demonstrated. Nitrogen-containing bisphosphonates inhibit specific protein and enzyme mechanisms within osteoclasts, thus interfering with their activity by triggering the cellular apoptosis and disrupting the cellular ultrastructure [118,119]. Several studies evidenced the anti-osteoporotic efficiency of HANP-based materials modified with alendronate [97,120], risedronate [121,122], and zoledronate [123,124]. By inhibiting osteoclast-mediated bone resorption, bisphosphonate-modified HA-based constructs—such as coatings [125,126], scaffolds [109,127], and injectable formulations [128,129]—determine a net improvement in osteogenic processes. Recently, HANPs loaded with salmon calcitonin polypeptide were proposed for the sublingual management of osteoporosis [130]. Promising results were also evidenced for HA-based biomaterials loaded with an anti-resorptive agent (denosumab) [131] or an anabolic agent (teriparatide) [132].

Following the development of the promising strontium ranelate (SR) (Protelos^®^/Protos^®^, Servier Laboratories, Surene, France) anti-osteoporotic drug, a variety of studies have been conducted, ranging from strontium (Sr) mapping in bones and teeth to investigating Sr incorporation into bone mineral (in particular, in the crystal surface and lattice) and a decrease in calcium content, and to evaluating Sr effects in synthetic HA. Sr has a dual positive effect during osteogenesis and bone remodeling, by boosting the development of pre-osteoblastic cells, while suppressing the generation and functionality of osteoclastic cells [133,134]. By gathering the distinctive advantages of HA and Sr, their composites represent a suitable choice for the controlled and targeted therapy of bone tissue [135,136,137].

Other studies revealed the significance of zinc (Zn)-enriched HA nanomaterials for the repair and regeneration of traumatic and osteoporotic bone tissues, as it has been evidenced that Zn addition is beneficial for enhanced osteogenesis and the prevention of osteoclast-mediated resorption [138,139,140].

Selenium (Se) is a vital micronutrient for human health, as it plays an important role in disease prevention and cellular pathophysiological balance maintenance. In this respect, Se-modified HA nanomaterials proved to be promising alternatives for bone tissue therapy, since the presence of Se synergistically determines enhanced cellular processes in healthy cells (adhesion, migration, proliferation, and osteogenic differentiation) [141,142] and significant apoptotic damage in cancerous cells [143,144].

In order to increase the structural integrity and to modulate interactions between the biological microenvironment and inorganic nanostructures, the surface modification of HA-based nanomaterials was explored [145,146]. The hydroxyl-abundant surface of HA is responsible for beneficial interactions with organic compounds, resulting in surface silanization and covalent bonding [147,148,149], immobilization and grafting [150,151,152]. Coupling natural [153,154] or synthetic [155,156] polymers onto the surface of HANPs has been shown to improve the NPs’ colloidal stability and mechanical qualities, together with their biofunctional outcomes. When used as bone-filling materials, such composite or hybrid structures can additionally act as active depots for the long-term release of pharmaceuticals, including drugs [157,158,159] and biomolecules [160,161,162].

Particular attention was oriented towards the fabrication of HANP-modified polymeric scaffolds, given the fact that a higher amount of nanoparticles triggers and accelerates the nucleation of biomimetic apatite, finally resulting in increased bone formation [146,163]. Designing HA/polymer constructs for bone tissue engineering requires fulfilling some essential aspects: (i) structural requirements: tissue-mimicking composition and architecture, adequate mechanical behavior, and highly porous interconnected structure, which are responsible for the osteoconductive and osteoinductive outcomes, as well as for proper cellular migration and normal development, oxygenation and nutrition, and vascularization; and (ii) biological requirements: biocompatibility, nontoxicity, non-immunogenicity, and biodegradability, which are vital aspects for enhanced osteogenesis and host integration [164,165,166].

Owing to their excellent biodegradability and nontoxicity, and particular resemblance with the natural extracellular matrix, natural polymers—such as proteins (e.g., collagen [109,167], gelatin [168,169], silk fibroin [170,171]) and polysaccharides (e.g., chitosan [172,173], cellulose [174,175], alginate [176,177])—are indisputable candidates for bone healing applications. The modification of such scaffolds with HA-based formulations represents an attractive strategy to overcome their intrinsic restrictions (improper mechanical properties, uncontrollable degradability, immunogenicity, and microbial contamination susceptibility).

In comparison with natural polymers, synthetic polyesters (e.g., polylactide (PLA) [178,179], poly(lactide-co-glycolide) (PLGA) [180,181], polycaprolactone (PCL) [182,183], and polyhydroxyalkanoates [184,185]) provide superior mechanical performance, increased chemical and structural stability, and tunable biodegradability. However, due to their intrinsic limitations (including hydrophobicity, slower degradation rate, and problematical metabolization/excretion of their byproducts), additional alterations are required to fabricate superior HA-modified bioactive scaffolds for bone healing.

**Figure 3:**
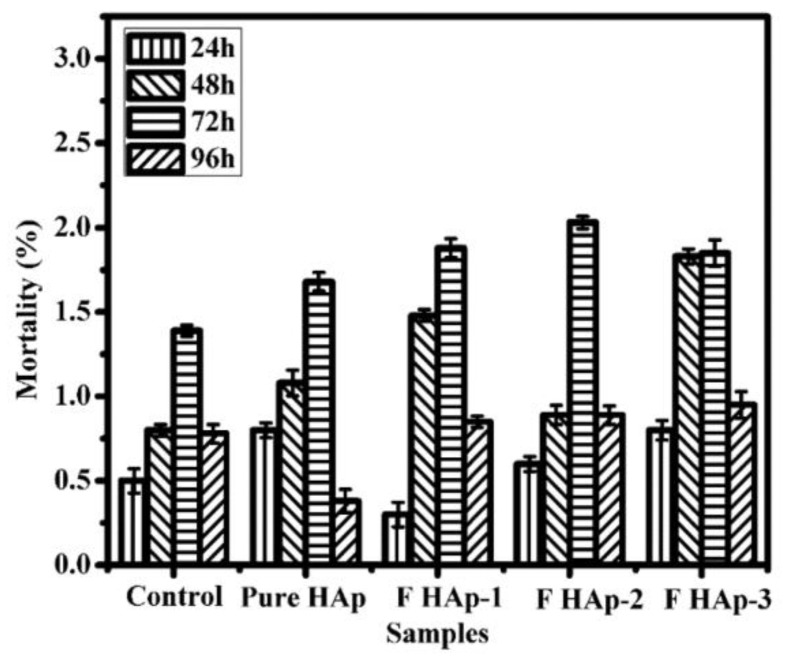
Quantitative representation of mortality (death rate, %) in zebrafish embryos treated with mesoporous fluoride-doped nano-hydroxyapatite (0.6, 1.2, and 3.2 at.% for FHAp-1, FHAp-2, and FHAp-3, respectively) with respect to time and concentration. The as-developed FHAp nanorods also exhibited important concentration-dependent antibacterial effects against *Pseudomonas aeruginosa* and *Bacillus subtilis*. See Ref. [192]. Reprinted from an open access source.

As particular representatives of HANPs, mesoporous nanostructures have gained great attention regarding the development of nanostructured platforms for the controlled therapy of bone tissue [186,187]. It has been demonstrated that mesoporous HANPs represent efficient nanocarriers for growth factors [188,189,190], antimicrobial ions [191,192] (Figure 3), antibiotics [193,194], and anti-tumor drugs [195,196,197], as a result of their uniform, accessible, and highly organized porous microstructure.

HANP-based therapeutic strategies have a lot of promise for bone tissue engineering, which represents a complex and challenging research field of modern biomedicine [198]. The characteristics of HA-based nanomaterials can be accurately optimized during the synthesis, in order to fabricate low-cost and performance-enhanced advanced biomaterials for therapeutic usage [199,200]. Nanofabrication techniques can provide precise control over the physicochemical and microstructural features of HANPs, which are mandatory for achieving spatial control over cell behavior, while imparting the necessary structural properties [201,202].

### 2.2. Bioactive Glass

Bioactive glasses (BGs), with their indisputable and versatile silica-based representatives, are amorphous solids which compositional and structural characteristics have been proved beneficial for the development of bioactive substitutes and platforms for bone tissue repair and regeneration [112,203]. BGs, firstly introduced in the early 1970s, opened up a new direction towards bone tissue therapy, as their intrinsic features (rapid and stable bonding with living tissues and surface-mediated reactions that encourage biomimetic apatite formation under physiological conditions) became prototypical requirements for designing bioactive materials [203,204].

An increased SiO_2_ content in silica-based BGs (of maximum 60%) is responsible for their strong bonding with the bone tissue (i.e., direct BG/bone interface, without fibrous connective tissue), which further provides enhanced interactions between surface-generated bone-like apatite layer and collagen fibers [203,205]. Besides the intrinsic osteostimulative characteristics of silicon-containing bioceramics [206,207], it has been evidenced that subsidiary ions released by the dissolution of BGs (calcium, sodium, and phosphorous) contribute to bone repair and regeneration by accelerating mineralization, stimulating cellular processes (proliferation, migration, and differentiation), and regulating the molecular mechanisms (protein and gene expression) involved in osteogenesis and angiogenesis [208,209,210]. The bioactivity of silica-based BGs can be further boosted by incorporating other ions that provide additional immunomodulatory and/or antimicrobial functions, such as magnesium [211,212], zinc [213,214], copper [215,216], silver [217,218], and strontium [219,220]. In addition to conventional BGs, phosphate-based [221,222,223] and borate-based [224,225,226] bioactive glasses have been explored for bone healing applications, but they require extensive composition-related control over their stability, dissolution, and biological activity [227,228].

**Figure 4:**
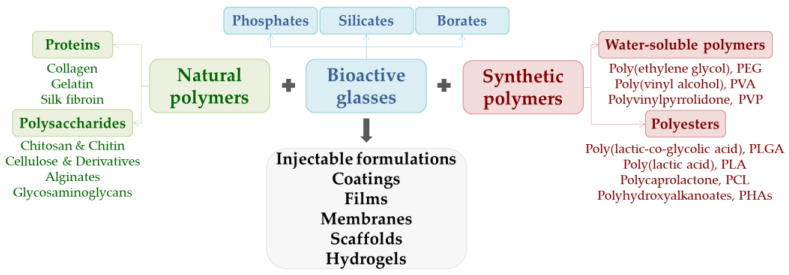
Schematic representation of bioactive glass/polymer composites in bone healing applications.

Besides encouraging stable bonding with host tissues, BGs also provide active sites for favorable interactions with polymers, both natural and synthetic, as briefly evidenced in Figure 4 [210,229,230]. BG/polymer composites possess advanced functionality in terms of mechanical performance, microstructure, reactivity, biodegradability, osteostimulation, and osteogenesis, thus representing suitable candidates for bone tissue engineering and regenerative medicine [210,229,231]. Since the key features of BGs, such as solubility and bioactivity, can be enhanced by changing the structure and particle size (at the nanoscale level), nanosized BGs are attractive and versatile fillers for biodegradable polymers when it comes to the fabrication of advanced composites for bone healing [232,233,234].

Because of their large specific surface area and rapid ion release rate in biological fluids, nanoscale bioactive glass particles display higher bioactivity than microscale bioactive glass particles. However, the conventional synthesis of bioactive glass nanoparticles (BGNPs) is challenging and problematic due to the difficulty of doping high amounts of calcium ions within the silica network, resulting in uneven distribution and low calcium content. Furthermore, BGNPs are often synthesized by using dilute solutions in order to avoid nanoparticle aggregation, thus reducing the production efficiency and raising the costs. Reactive flash nanoprecipitation [235] and ultrasound-assisted sol–gel [236,237] were proposed as successful alternatives for the traditional sol–gel synthesis of BGNPs, resulting in particles with a more homogenous calcium-enriched composition, smaller size and narrower size dispersion, and superior bioactivity.

The ability to incorporate active ions within their composition is a significant advantage of BGNPs over other inorganic nanoparticles, as the release of such ions during dissolution opens up a world of possibilities for enhancing the biofunctional outcome of nanoengineered composites. Doping BGs with antimicrobial ions represents a promising strategy for the fabrication of bone fillers or bone grafts that can allow bone repair and regeneration without the risk of post-implant infections [238,239]. Therefore, the potential use of BGs doped with zinc (Zn)—Zn-BGs—was thoroughly investigated [240], as the presence of Zn determined antibacterial effects, and also contributed to enhanced mineralization and osteogenic activity [241,242]. Beneficial effects with respect to in vitro mineralization, cellular development, and antimicrobial efficiency, were also evidenced in the case of silver (Ag)-doped BGs (Ag-BGs) [218,243].

Despite the promising results reported in BGNP-based composites and devices, significant efforts must be made in order to fully explore and beneficially revalue the biological potential of such nanomaterials, as there is a lack of data regarding the long-term in vivo safety and performance of BGNPs [244,245].

In comparison with conventional BGNPs, mesoporous bioactive glass nanoparticles (MBGNPs) provide additional advantages regarding the microstructure-related ability to load and release therapeutic agents, representing multifunctional platforms for bone healing applications. MBGNPs are usually obtained by sol–gel-mediated protocols [246,247], and their versatile composition enable the incorporation of different therapeutic compounds, including copper [248,249], silver [218,250], and zinc [251,252] for antimicrobial effects, osteogenic activity, and immunomodulation; strontium for pro-osteogenic and pro-angiogenic effects [253,254]; cerium and gallium for antibacterial activity and bioactivity [255,256]; cobalt [257], iron [258], selenium [259], and tellurium [260] for anti-cancer effects.

**Figure 5:**
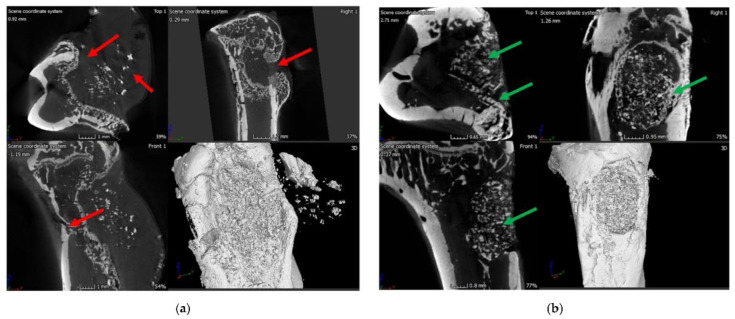
Micro-computed tomography (μ-CT) images of the infected rat tibia (control group), evidencing signs of infection at 8 weeks: narrowing of marrow space, presence of puss-filled fibrous capsule, sinus tract, and deformed bone with ectopic bone growth (red arrows) (**a**). μ-CT images of the infected rat tibia treated with vancomycin-loaded polymer/BG bone void-filling putty at 8 weeks post-implantation, evidencing signs of healing bone, as well as the formation of cortical and cancellous bone in the drilling space (green arrows) (**b**). The as-developed scaffolds also determined the in vivo eradication of *Staphylococcus aureus*. See Ref. [262]. Reprinted from an open access source.

In addition to their intrinsic capabilities (osteoconductive, osteoinductive, and angiogenic effects), MBGNPs represent attractive nanocarriers for the controlled and targeted delivery of antibiotics [261,262] (Figure 5), anti-osteoporotic drugs [263,264], chemotherapeutic agents [265,266], and biomolecules [267,268], thus providing an unrivaled and prospective edge towards designing innovative smart materials for bone tissue therapy [246,269,270].

## 3. Oxide Nanoparticles

### 3.1. Mesoporous Silica

Silicon (Si) is naturally found in the human body, and it has a regulatory role during the normal development of the skeleton and connective tissues, and also has beneficial effects during collagen synthesis and matrix mineralization [271,272]. Besides representing a major source of Si ions, silica (SiO_2_)-based nanomaterials—especially mesoporous silica nanoparticles (MSNs)—provide attractive and tunable characteristics for biomedical applications, including drug/biomolecule delivery systems [273,274,275], tissue engineering [276,277,278], regenerative medicine [279,280,281], and cancer therapy [282,283,284].

A large surface area and pore volume ratio, adjustable particle size, well-structured internal and external porosity, uniform and controllable pore size, impressive surface functionalization, and intrinsic biocompatibility, represent the key features of MSNs used for the fabrication of therapeutic biomaterials and devices [285,286,287]. The porosity characteristics of MSNs can be explored for loading various therapeutics, including biomolecules, soluble and insoluble drugs, targeting molecular drugs, and imaging agents, as well as their different combinations, which may be simultaneously released within the impaired tissues to achieve improved local concentration and synergistic drug therapy and diagnostics (theranostics) [288,289,290]. Moreover, the pore-opening gating mechanisms distinguished in MSNs provide indisputable advantages over the controlled release of the therapeutic cargo in response to internal (e.g., weakly acidic local microenvironment, cancer-overexpressed enzymes, or other biomolecules) and external (e.g., light, ultrasound, and magnetic field exposure) stimuli [291,292].

Although MSNs represent one of the most appealing nanomaterials for the fabrication of performance-enhanced constructs for bone healing applications, some critical parameters must be considered in order to achieve the desired therapeutic effects. By optimizing the synthesis parameters (such as the type of silica precursor, the pH and temperature during the reaction, and the type and concentration of surfactant), the size, morphology, and porosity of MSNs can be modified [293,294]. Conventional and modified sol–gel, evaporation-induced self-assembly, and core-templating synthesis (in the case of hollow MSNs) represent the most explored strategies for fabricating MSNs with controllable particle and pore sizes [295,296].

**Figure 6:**
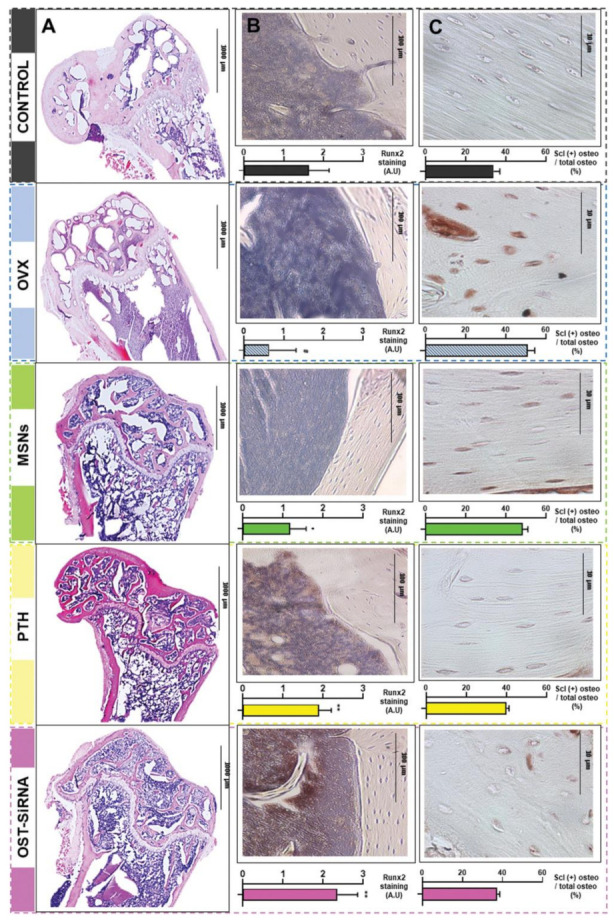
Histological analysis and immunostaining in the femur of osteoporotic mice at 3 weeks post-treatment with mesoporous silica nanoparticles (MSNs) grafted with alendronate-modified poly(ethylene glycol) and poly(ethylene imine) (MSNs-PA@PEI), parathyroid hormone (PTH), and MSNs-PA@PEI loaded with osteostatin and sclerostin-encoding plasmid (OST-SiRNA), evidencing: representative micrographs of different femur histological sections after hematoxylin/eosin and Masson–Goldner trichrome staining (**A**); representative Runx2 immunostaining in mice femurs, revealed by the abundant positivity (brown stains) for the transcription factor in cells after PTH or OST-siRNA treatments (**B**); total and sclerostin-positive osteocytes in the cortical femur (**C**). Data are represented as mean ± standard error of mean of five independent mice (*n* = 5), and the statistical significance is indicated as # *p* < 0.001 vs. control, * *p* < 0.05 vs. ovariectomized mice (OVX), and ** *p* < 0.001 vs. OVX. See Ref. [302]. Reprinted from an open access source.

Vital events involved in bone repair and regeneration, including cellular proliferation and differentiation, bone matrix mineralization, osteoinduction, and osteogenesis, can all be triggered or boosted by means of Si-enriched nanosized and nanostructured materials [297,298]. Through their modulatory effects on the specific molecular complexes involved in bone homeostasis, MSNs stimulate pro-osteoblastic action and mineralization, induce osteogenic differentiation and angiogenesis, and inhibit osteoclasts, thus influencing the osteoblast/osteoclast ratio [47,299,300]. Moreover, the bone healing process can be promoted or accelerated by loading osteoinductive proteins [301,302] and related encoding peptides [303] or encoding plasmids [302,304] (Figure 6) within MSN-based formulations. Besides their intrinsic bioactivity, MSNs exhibit impressive opportunities for fabricating multifunctional platforms for bone healing therapy, as their distinctive open porous microstructure enables the incorporation and release of various therapeutic molecules [305,306].

**Figure 7:**
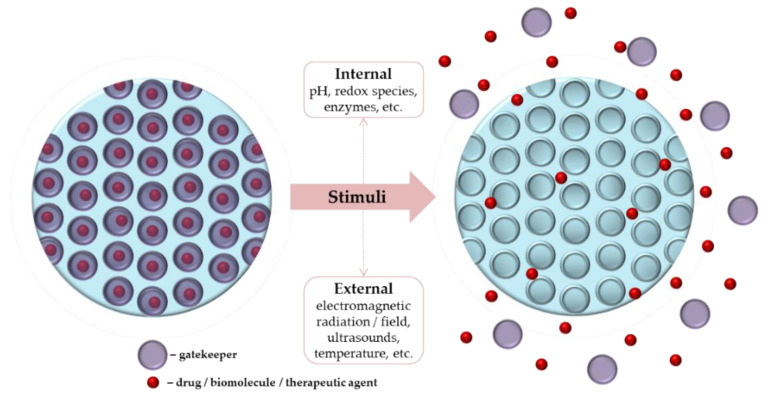
Schematic representation of stimuli-responsive mesoporous silica nanoparticles (MSNs).

MSNs possess an impressive ability to transport therapeutic biomolecules and active targeting molecules into impaired bone cells, thus representing attractive multifunctional platforms for bone tissue therapy. In addition, the premature and non-specific release of the therapeutic cargo can be limited or even eliminated by using gatekeepers (e.g., nucleotides, natural or synthetic polymers, and metallic nanoparticles) that block pores and provide on-demand pore opening and closing in response to certain stimuli (Figure 7) [307,308,309]. The as-fabricated MSN-based platforms can act as active carriers for chemo drugs, anti-resorptive agents, antibiotics, and genes, providing targeted and controlled therapy for bone-related pathologies, in addition to their intrinsic bone healing effects [307,310].

The incorporation of MSNs within three-dimensional nanoengineered networks provides tremendous possibilities for the specific and selective management of bone infection and bone cancer [310,311,312]. Besides their compositional and structural resemblance with the natural tissue, artificial scaffolds exhibit increased loading efficiency and modulated release of pristine or nanosystem-conjugated drugs/biomolecules [313].

MSN-based nanosystems have been evaluated as efficient loading/releasing vehicles for several antibiotics [314,315,316]. Moreover, composite scaffolds incorporating cephalexin-loaded MSNs [276] and vancomycin-loaded MSNs [317] proved to represent promising candidates for the local treatment of bone infection, while promoting bone healing.

The specific and selective management of bone cancer can be achieved with MSNs-based carriers that target particular receptors that are overexpressed in cancer cells [318,319,320]. The cellular uptake of such nanostructures can also be improved by considering particular features of the tumor microenvironment [321,322] or by altering the intrinsic regulatory mechanisms of highly metabolically active cancerous cells [323,324,325]. Moreover, the versatile functionality of MSNs can also be explored for developing unconventional anti-cancer strategies by means of non-radioactive and controlled alternatives mediated by nanostructures conjugated with active targeting molecules and loaded with reduced drug concentrations or/and sono/photosensitizers [326,327,328].

### 3.2. Iron Oxide

Magnetic nanoparticles (MNPs) possess magnetic, semiconductor, nontoxic, and bioactive properties all at once, and play a critical role in the progress of modern biomedicine, with particular outcomes towards the specific and selective therapy of bone tissue [329,330]. The biomedical versatility of iron oxide nanoparticles, as particular representatives of the magneto-responsive nanostructures, relies on their multifunctional size-related features, such as intrinsic biocompatibility and biodegradability, surface chemistry and reactivity, and tunable magnetism (with particular superparamagnetic behavior for ultra-small MNPs) [331,332].

Besides their intrinsic size-governed anti-infective [333,334,335] and anti-tumor effects [336,337,338], the surface modification of MNPs with inorganic capping layers [339,340,341], therapeutic molecules [342,343,344], and biomolecule-conjugated macromolecule layers [345,346,347] paves the way towards the fabrication of accurate and efficient strategies for bone healing. The impressive functionalization potential of superparamagnetic iron oxide nanoparticles (SPIONs) enables the fabrication of active platforms for bone repair and regeneration, as well as for bone infection and cancer. Such magnetic nanostructures can act as active vehicles and therapeutic enhancers for their cargo, but their functionality can be extended by means of external triggers (electromagnetic radiation and fields), which represent the leading advantage of MNP-based biomedicine [348,349,350].

Following their exposure to an alternating magnetic field, MNPs undergo important magnetic relaxation, as their magnetic moment (given by unpaired spin electrons in the outermost electron shell) rapidly flips its orientation between two stable states, but they also can undergo physical rotation and circumstantial collisions, finally resulting in converting the external energy into heat [351,352]. This peculiar behavior of MNPs gives them an impressive potential for the local thermally-induced alteration of pathological cells by means of magnetic hyperthermia, which is being extensively investigated for cancer management [353,354]. Moreover, if therapeutic agents are conjugated to MNPs, their local release can be externally triggered and controlled. Even if the clinical application of magnetically targeted therapy by means of magnetized medications still requires regulatory protocols [355,356], the preclinical evaluation of SPION-mediated bone cancer therapy is of great interest. Besides acting as mechanical reinforcements for polymeric scaffolds, SPIONs contribute to the normal development of bone cells and promote the mineralization process and osteogenic activity [357,358,359], and also promote the in vivo bone repair and regeneration [359,360,361]. In addition to their ability to generate localized hyperthermia while avoiding the impairment of surrounding normal tissues when combined with SPIONs, it has been reported that magnetic fields are beneficial for promoting the osteogenic activity of progenitor cells. Magnetic fields regulate the cellular uptake of SPIONs via stem cells and preosteoblasts and promote their osteogenic differentiation and bone matrix mineralization, and also contribute to their proliferation, migration, and organization inside scaffolds [362,363], finally resulting in magnetically guided osteogenesis and angiogenesis [364,365,366]. SPION-loaded constructs (e.g., porous inorganic scaffolds, polymer sponges, and hydrogels) and external magnetic fields synergistically act to provide successful therapeutic alternatives for bone healing [329,367].

**Figure 8:**
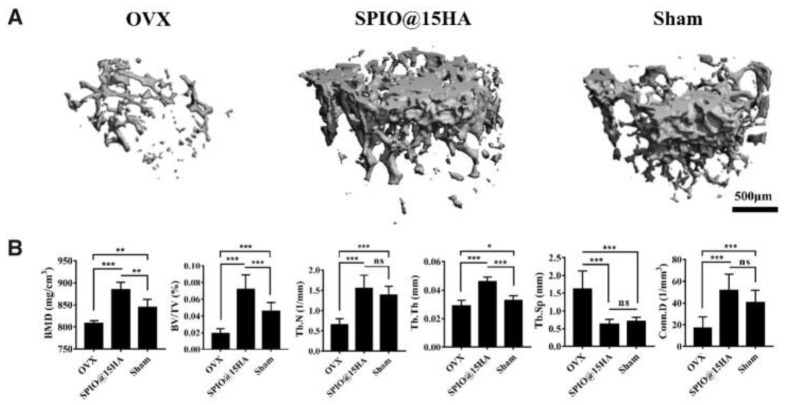
Three-dimensional μ-CT reconstruction images of trabecular bone in ovariectomized mice (OVX), OVX treated with hydroxyapatite-coated superparamagnetic iron oxide nanocomposites (SPIO@15HA) and sham group (**A**), and trabecular bone mass parameters (**B**), evidenced after 3 months post-injection. BMD—bone mineral density, BV/TV—bone volume fractions, Tb.N—trabecular number, Tb.Th—trabecular thickness, Tb.Sp—trabecular spacing, Conn.D—connectivity density. Data are expressed as mean ± standard deviation of seven independent mice (*n* = 7), ns means no significance, and the statistical significance is indicated as * *p*  <  0.05, ** *p*  <  0.01, and *** *p*  <  0.001. See Ref. [370]. Reprinted from an open access source.

Magneto-responsive HA/SPIONs composites have been investigated particularly for bone healing applications owing to their synergistic effects. HA/SPIONs formulations exhibit intrinsic antimicrobial effects [368,369] while promoting osteogenesis and neovascularization and inhibiting osteoclastogenesis [370,371,372] (Figure 8). In addition, their drug carrier ability opens the way for efficient and accelerated infection-free bone repair [97,373].

Given the extensive use of metallic implants in the clinical restoration and replacement of bone tissue, an attractive nanotechnology-derived approach consists of enhancing their bioactivity and osteogenic activity using surface coatings [374,375,376]. It has been reported that the incorporation of SPIONs within HA [377,378] or polymer [379,380] coatings leads to significant improvements in the wettability and corrosion resistance of titanium-based biomaterials, and also enhanced apatite-forming ability and cellular events. As the direct interactions between SPIONs and therapeutic agents determine the formation of highly stable nanosystems with potentiated therapeutic effects, such nanostructures have been extensively investigated with respect to the development of new pharmaceuticals [35,381,382]. The therapeutic outcome of metallic implants can be achieved by means of synthetic polyester coatings embedded with MNPs conjugated with natural antimicrobial extracts [383], electroactive polymer coatings embedded with antibiotic-functionalized MNPs [379,380], and chemo drug-loaded SPIONs/cyclodextrin coatings [384].

### 3.3. Other Oxides

The therapeutic implications of other oxide nanoparticles in bone healing applications have been also explored [385,386]. For instance, magnesium oxide (MgO) and zinc oxide (ZnO) nanoparticles have been investigated for the fabrication of functional bone substitutes [387,388]. MgO and ZnO NPs exert strong antimicrobial and anti-biofilm activity [389,390], and also antioxidant effects [391,392], making them suitable candidates for boosting the performance of HA-based substitutes [393,394,395,396].

Following their dissolution, MgO NPs provide mineral nutrients that are essential for most biological processes, including new bone formation, by promoting osteogenic proliferation and differentiation and bone-like mineral deposition [397,398,399]. By exerting positive immunomodulatory effects, MgO NPs indirectly suppress the activity of osteoclasts [400]. Besides acting as mechanical reinforcements for polymeric scaffolds, MgO NPs also modulate their hydrophilicity and degradation, whilst the polymeric matrix enables the gradual release of therapeutic ions, finally resulting in enhancing the bone healing ability of such composites [401,402].

Given the fact that an imbalance in the normal zinc deposits and cellular zinc homeostasis may occur after bone tissue injuries (as the human skeleton is a major source of zinc), producing zinc-enriched substitutes is of great importance for bone healing and normal skeletal development [403,404]. ZnO NPs synergistically act on the bone cells involved in bone formation and remodeling by inducing osteogenic effects [405,406] and modulating the osteoclastogenic events [407,408]. The oxidative events induced by ZnO NPs (mediated by free zinc ions and reactive oxygen species) can be further explored for bone tissue regeneration and bone cancer therapy through their pro-angiogenic [409,410] and anti-angiogenic [411,412] properties, respectively.

**Figure 9:**
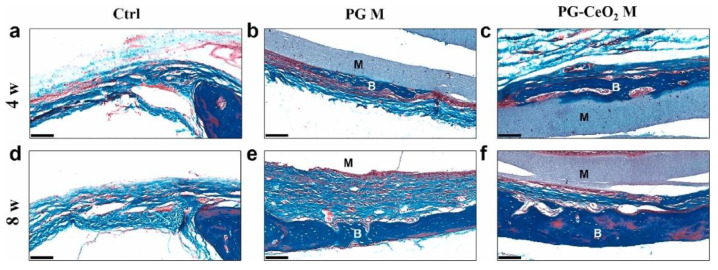
Histological analysis of rat cranial defects treated with bare and nano-ceria-loaded polycaprolactone/gelatin membranes (PG M and PG-CeO_2_ M, respectively) for 4 and 8 weeks (w), evidenced by Masson’s trichrome staining. Control group (**a**,**d**), PG M group (**b**,**e**), and PG-CeO_2_ M group (**c**,**f**). M—membrane, B—bone, scale bar—100 μm. See Ref. [417]. Reprinted from an open access source.

It has been evidenced that cerium oxide (ceria) NPs stimulate the osteogenic differentiation of stem cells and regulate bone mineralization, and also exhibit antioxidant effects (which are beneficial for limiting the oxidative events that may occur during slow bone regeneration and bone-related inflammatory pathologies) [413,414,415]. Nano-ceria also modulates the angiogenesis process of ceramic and polymeric biomaterials following their implantation, resulting in accelerated new bone formation [416,417] (Figure 9). Moreover, the stimuli-responsive ability of ceria NPs [418,419], together with their radio-protective effects [420,421] and intrinsic antibacterial effects (evidenced against extracellular and intracellular pathogens) [422,423], open up new ways for the efficient treatment of bone diseases.

Recently, hollow manganese oxide NPs were proposed as efficient platforms for the immunotherapy of osteosarcoma, with their additional tumor-targeting ability and imaging-guided drug delivery [424]. These oxide nanoparticles exhibit important osteogenic activity and bone-forming ability [425,426], while their excellent antioxidant effects proved to be beneficial for the management of osteoarthritis [427,428].

A significant improvement in the mechanical behavior and thermal stability of polymeric biomaterials has been evidenced after the incorporation of titanium oxide (titania) NPs, with such nanostructured platforms being proposed for the long-term use in bone regeneration [429,430]. The efficiency of nano-titania on osteoblast/osteoclast homeostasis [431,432] and collagen deposition (by inducing the secretion of biomolecules that actively regulate bone repair) [433], without affecting the differentiation and mineralization of osteoblasts [433,434], has been reported.

## 4. Metallic Nanoparticles

This review also covers the implications of metal-based nanoparticles in bone tissue therapy. Owing to their peculiar nanosize-related characteristics, which include biomechanics and thermochemistry, stability and optical behavior, reduced toxicity and good biocompatibility, proliferative and intrinsic osteogenic potential, cellular development modulation, and intrinsic antimicrobial and anti-cancer effects, metallic NPs are versatile candidates for bone healing applications [385,435].

### 4.1. Gold

Gold nanoparticles (AuNPs) are biocompatible and inert nanosized structures with high monodispersity, electroconductivity, and excellent optical properties [436,437]. The impressive use of AuNPs in modern biomedicine relies on their highly remarkable surface functionalization potential, and includes targeted therapeutic formulations (drug, macromolecule, peptide, protein, and gene delivery), biomedical imaging and diagnosis (biodetection and biosensing), and complex therapy (photothermal, photodynamic, and radiation therapy) [438,439,440].

In relation to bone healing therapy, it has been evidenced that AuNPs exhibit intrinsic osteogenic effects (by promoting the differentiation of pluripotent cells and biomimetic apatite formation) [441,442], inhibit osteoclastogenesis [443,444], and accelerate de novo bone formation [445,446]. Several molecular mechanisms were proposed for AuNP-mediated osteogenic differentiation [439,447]. Stem cells may undergo osteogenic differentiation in response to extracellular AuNPs (physical and/or chemical modification of the microenvironment) and intracellular AuNPs (mechanical stress) by means of the integrin-mediated signaling pathway [448,449], transcellular pathway [441,450], and autophagy [442,451]. It has also been evidenced that the osteogenic ability of AuNPs is strongly related to their concentration [452], size [445], and shape [453].

What is more, AuNPs also exhibit important antimicrobial [454,455] and anti-cancer [456,457] activity. By considering the multifunctional therapeutic effects of AuNPs, and also their impressive functionalization versatility, substantial efforts have been oriented towards the fabrication of AuNP-embedded composites and complex formulations for bone repair and regeneration [439,458]. Moreover, given their peculiar electrical and optical behavior, AuNPs have been explored for the targeted and controlled management of bone infections and bone cancers [385,459].

### 4.2. Silver

Silver nanoparticles (AgNPs) are one of the most explored nanosized noble metals in modern industrial and biomedical applications, owing to their intrinsic catalytic effect, chemical stability, good electrical conductivity, optical behavior, and versatile biological activity [460,461]. In its ionic, metallic, and nanoparticulate forms, silver has been extensively used as an antibacterial agent [462,463]. The particular anti-pathogenic effects of nano-silver have been assigned to their ability to adhere to bacterial cell walls and produce oxidative stress, resulting in the bacterial cell wall and membrane impairment and subsequent cytoplasmic leakage, and the denaturation of bacterial macromolecules and alteration of vital cellular processes, respectively [464,465,466]. Silver ions released by AgNPs mediate bacterial death by impairing the peptidoglycan component of cell walls, hindering bacterial protein synthesis and obstructing replication signals and energy-dependent survival processes by binding to nucleic acids [467,468].

**Figure 10:**
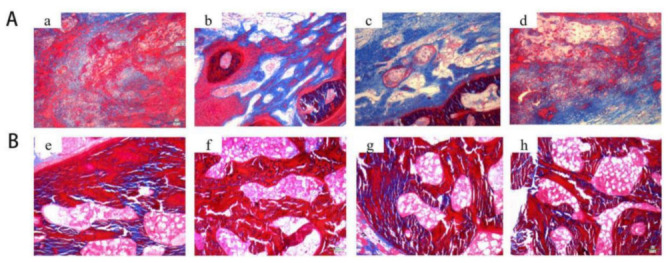
Histological analysis of rabbit skull defects treated with bare and nano-silver-loaded gelatin/alginate scaffolds (Gel/Alg and AgNP–Gel/Alg, respectively) for 4 weeks (**A**) and 8 weeks (**B**), evidenced by Masson staining (100×). Gel/Alg group (**a**,**e**); 200 μM AgNP–Gel/Alg group (**b**,**f**); 400 μM AgNP–Gel/Alg group (**c**,**g**); 600 μM AgNP–Gel/Alg group (**d**,**h**). See Ref. [475]. Reprinted from an open access source.

In the realm of orthopedics and dentistry, where the infection susceptibility of implanted devices is a continuous danger, the clinical potential of nano-silver is of special interest [469,470,471]. Since AgNPs stimulate osteogenesis and inhibit osteoclastogenesis [472,473], their use in bone healing applications gives rise to multifunctional platforms, and such nanostructures can be used to induce or potentiate the antimicrobial effects of nanoengineered constructs and clinically used devices, while stimulating the osteogenic activity [474,475,476] (Figure 10).

Modifying surface coatings with AgNPs represents an attractive strategy to enhance the bioactivity and osseointegration of metallic implants used in orthopedics and orthodontics. The use of AgNPs in oxide and non-oxide ceramic coatings can minimize the infection susceptibility of metallic implants by modulating the coating’s resistance to bacterial contamination and colonization, and exerting broad-spectrum antibacterial effects, while they maintain or improve their beneficial effects on osteogenic activity [477,478,479]. Furthermore, embedding AgNPs within polymer coatings represents an attractive strategy to generate antimicrobial surfaces for bone implants, with the additional osteogenic ability and bone-forming potential [480,481,482]. Such nanostructured layers act synergistically, as the inorganic nanosystems locally exert their antimicrobial effects, while the polymer matrix prevents their agglomeration, protects them from external damage, and provides an active carrier for their local release [483,484], and also prevents AgNP-mediated local tissue reactions [485].

In bone healing therapy, particular attention was oriented towards the incorporation of AgNPs within biomimetic constructs, such as HA-based coatings and polymer/HA scaffolds. Besides their anti-pathogenic effects, such nanomaterials proved to be beneficial substrates for mineralization and osteogenic differentiation, finally resulting in enhanced osseointegration of the metallic implants [486,487,488] and functional bone substitutes [489,490], respectively.

AgNPs exhibit nanosize-governed intrinsic anti-cancer activity (as evidenced against various cancer types) [491,492], and they also exert potentiating effects on chemotherapeutic agents [493,494] and alter tumor angiogenesis [495,496]. The local release of silver ions after cellular uptake determines cellular oxidative damage, impairment of cellular substructures, and subsequent apoptosis and necrosis [497,498,499,500]. The efficiency of AgNP-based formulations on bone cancers has been investigated against osteosarcoma [501,502,503], rhabdomyosarcoma [504,505], Ewing’s sarcoma [506], and chondrosarcoma [507].

### 4.3. Copper

Copper (Cu) is one essential trace element found in the human body that has a vital role in the cellular events that maintain the normal function of bones, blood vessels, and nerves, and it also contributes to wound healing speed, antioxidant defense, and immune function [508,509,510].

Copper deficiency has been linked with several disorders that mostly affect the connective and bone tissues. Cu plays a vital role in bone metabolism, and its lack may cause bone anomalies and deformities [386,511]. It has been evidenced that Cu deficiency leads to an inhibited activity of the oxidases (enzymes which normal function requires trace element cofactors) that are involved in collagen synthesis and vitamin D activation, thus resulting in the increased solubility of bone collagen, damaged peptide chain connections, impaired bone collagen stability, and reduced bone strength [512,513].

Given its beneficial role in bone metabolism, the use of Cu-based formulations—with particular emphasis on metallic ions and nanoparticles—is of great interest for bone healing applications. Following their incorporation or immobilization within different materials, copper nanoparticles (CuNPs) exhibit increased chemical stability and a self-tuned ability to gradually release the metallic ions without affecting the stability of matrix materials [514,515].

Furthermore, all forms of copper, including ions, nanoparticles, and alloys, possess excellent antibacterial properties, alongside osteogenic and angiogenic effects [508,516]. In a similar way to AgNPs, the antibacterial action of CuNPs relies on the conjunction between the nanosize-related impairment of cellular structures and metallic ion-mediated events (oxidative damage, obstructed protein synthesis, inhibited replication, and altered cellular survival processes) [517,518,519]. CuNPs also exhibit powerful antioxidant action (thus neutralizing free radicals and preventing cell damage) [520,521] and anti-cancer activity [522,523].

Even if substantial studies must be performed to properly and accurately revalue their therapeutic potential [524,525], Cu-based formulations represent multifaceted candidates for bone tissue therapy, as evidenced by their bone healing ability (enhanced mineralization, osteogenesis and angiogenesis, and modulated osteoclastogenesis) [526,527,528], extended antibacterial activity [515,529,530], and anti-tumor efficiency [531,532]. CuNPs have also been investigated with respect to dental applications, as efficient antimicrobials for denture base resins [533], endodontic treatment [534], and periodontitis management [535].

## 5. Conclusions and Perspectives

Designing successful devices and substitutes for bone therapy still represents a challenge for modern biomedicine, as it implies the accurate understanding of bone pathophysiology, the proper selection of biomaterials and fabrication protocols, and maximal therapeutic efficiency.

Nanoparticle-based biomaterials and biotechnologies have been lately validated as viable alternatives to traditional scaffolding protocols. In particular, bioceramic, oxide, and metallic nanoparticles demonstrated impressive therapeutic outcomes for bone repair and regeneration, and also for bone pathologies management.

Owing to their bioactivity, biomimetic composition, and good incorporation within the natural bone structure, bioceramic nanoparticles represent the best choice for reparative and regenerative bone therapy. Their acknowledged cytocompatibility and beneficial interactions with living tissues can be explored in conjunction with polymeric constructs and other inorganic (ions, nanoparticles, alloys, and composites) or organic substances (drugs and biomolecules) in order to fabricate bone-mimicking platforms for the specific and selective management of bone pathologies.

Even if substantial efforts should be made to completely understand and finely tune the implications of oxide and metallic nanoparticles in bone healing, their functional versatility (as nanocarriers, imaging agents, and sensitizers) and intrinsic therapeutic activity are impressive. Such peculiar characteristics pave the way towards the development of multifunctional bone substitutes, including platforms for targeted and localized drug delivery (antimicrobial, anti-inflammatory, anti-resorptive, and anti-cancer therapy), specific and selective detection and diagnosis, and effective combined therapy.

Besides being active components for bone processes (contributing with their osteoconductive, osteoinductive, and osteogenic effects), the previously discussed inorganic nanomaterials exhibit additional biological activities (antimicrobial, antioxidant, immunomodulatory, anti-resorptive, and anti-cancer). The nanosize-governed surface chemistry of these nanoparticles provides active sites for the conjugation of various therapeutic agents (e.g., ions, nanostructures, drugs, biomolecules, and nucleic acids), and also enables their immobilization or incorporation into more complex constructs, finally resulting in the development of versatile and performance-enhanced candidates for bone healing applications.
